# Tribbles Pseudokinases in Colorectal Cancer

**DOI:** 10.3390/cancers13112825

**Published:** 2021-06-05

**Authors:** Bibiana I. Ferreira, Bruno Santos, Wolfgang Link, Ana Luísa De Sousa-Coelho

**Affiliations:** 1 Centre for Biomedical Research (CBMR), Campus of Gambelas, Universidade do Algarve, 8005-139 Faro, Portugal; biferreira@ualg.pt (B.I.F.); a66853@ualg.pt (B.S.); 2 Algarve Biomedical Center (ABC), Campus de Gambelas, Universidade do Algarve, 8005-139 Faro, Portugal; 3 Faculdade de Medicina e Ciências Biomédicas (FMCB), Campus de Gambelas, Universidade do Algarve, 8005-139 Faro, Portugal; 4 Serviço de Anatomia Patológica, Centro Hospital Universitário do Algarve (CHUA), 8000-386 Faro, Portugal; 5 Instituto de Investigaciones Biomédicas “Alberto Sols” (CSIC-UAM), Arturo Duperier 4, 28029 Madrid, Spain; 6 Escola Superior de Saúde (ESS), Campus de Gambelas, Universidade do Algarve, 8005-139 Faro, Portugal

**Keywords:** tribbles, colon cancer, oncogene, colorectal cancer, pseudokinase, biomarker, gene expression, genomic amplification, pharmacological target

## Abstract

**Simple Summary:**

The Tribbles family of pseudokinases controls a wide number of processes during cancer on-set and progression. However, the exact contribution of each of the three family members is still to be defined. Their functions appear to be context-dependent as they can act as oncogenes or tumor suppressor genes. They act as scaffolds modulating the activity of several signaling pathways involved in different cellular processes. In this review, we discuss the state-of-knowledge for *TRIB1*, *TRIB2* and *TRIB3* in the development and progression of colorectal cancer. We take a perspective look at the role of Tribbles proteins as potential biomarkers and therapeutic targets.

**Abstract:**

The Tribbles family of pseudokinases controls a wide number of processes during cancer on-set and progression. However, the exact contribution of each of the three family members is still to be defined. Their function appears to be context-dependent as they can act as oncogenes or tumor suppressor genes. They act as scaffolds modulating the activity of several signaling pathways involved in different cellular processes. In this review, we discuss the state-of-knowledge for *TRIB1*, *TRIB2* and *TRIB3* in the development and progression of colorectal cancer. We take a perspective look at the role of Tribbles proteins as potential biomarkers and therapeutic targets. Specifically, we chronologically systematized all available articles since 2003 until 2020, for which Tribbles were associated with colorectal cancer human samples or cell lines. Herein, we discuss: (1) Tribbles amplification and overexpression; (2) the clinical significance of Tribbles overexpression; (3) upstream Tribbles gene and protein expression regulation; (4) Tribbles pharmacological modulation; (5) genetic modulation of Tribbles; and (6) downstream mechanisms regulated by Tribbles; establishing a comprehensive timeline, essential to better consolidate the current knowledge of Tribbles’ role in colorectal cancer.

## 1. Introduction

Colorectal cancer (CRC) refers to a malignant tumor that arises from the colon or rectum epithelium. They are often referred together due to their many features in common. Worldwide, CRC was the third most common malignancy (1,931,590 cases; 10%) and the second most frequent cause of cancer deaths (935,173 cases; 9.4%), in both genders and all ages, in 2020 [1]. The fact that mortality is approximately half the incidence suggests a relatively good prognosis, which is closely related to improvements in cancer treatment and management. Since survival is highly dependent on the stage of cancer at diagnosis, early detection stands out as one of the best prognosis factors. The majority of cases of this highly heterogeneous disease appear between 50 and 75 years old, being more frequent in men than in women [2]. Risk factors are still unclear, but evidence supports the association of poor diet, lack of physical activity, smoking and an increasing prevalence of overweight, obesity, and type 2 diabetes with CRC [2].

Sporadic cases are the majority of CRC, while germline mutations and hereditary syndromes account for only 6 to 10% of all cases [3]. Two molecular pathways are traditionally associated with sporadic CRC: the adenoma–carcinoma, or chromosomal instability, pathway (70–75%), and the serrated pathway (25–30%) [2]. In the adenoma–carcinoma pathway, CRC evolves from non-malignant precursor lesions called adenomas, for a period of at least 10 years [4]. This process begins with the activation of Wnt signaling due to the loss of the tumor suppressor APC (Adenomatous polyposis coli) through inactivating mutations. Over the years, a series of mutations start to accumulate. KRAS oncogene mutation arises preferentially in early steps, while TP53, SMAD4, PIK3C, and PTEN mutations occur at later phases [5]. For the serrated pathway, the earliest event on sessile serrated adenomas is thought to be the activation of the MAPK pathway through BRAF oncogene mutation leading to a global methylation of CpG islands and consequently silencing of the mismatch repair gene MLH1 or the cyclin-dependent kinase inhibitor p16. In traditional serrated adenomas that comprise less than 1% of all serrated lesions, MAPK pathway activation is more frequently associated with mutation of the KRAS oncogene, although BRAF mutation may also occur [6,7].

Although driver mutations are recognized, over time several of these pathways may overlap or interact. Upon Wnt activation, β-catenin accumulates and translocates into the nucleus where it binds TCF4 to promote the transcription of *Jun*, *c-Myc* or *CyclinD-1*, promoting tumor progression. On the other hand, *APC* mutation promotes Wnt signal transduction by stabilization of β-catenin [8,9,10]. Overexpression of amplified genes like *MYC* and *MET* can also occur through double minute (DM) chromosomes (round-circle, acentric double-strand extra-chromosome DNA that usually exist in pairs). The formation of DM chromosomes is usually regarded as an important sign of genome instability and occurs in 2.6% of CRC [11,12].

As increased EGFR expression has been observed during colorectal carcinogenesis, targeted therapies with monoclonal antibodies such as cetuximab and panitumumab which prevent EGFR activation are commonly used in late-stage CRC. However, mutations in *KRAS*, *NRAS*, and *BRAF*, and amplification of *ERBB2* and *MET*, represent mechanisms of primary and secondary resistance to anti-EGFR therapy. In fact, only patients with wild-type *RAS* benefit from anti-EGFR therapy. Nonetheless, RAS-BRAF wild type CRC is also associated with resistance to anti-EGFR treatment in the presence of high c-MYC levels [13,14,15,16]. Targeting specific signaling pathways using multiple agents is the best option to achieve a better therapeutic outcome. Clinical trials have shown promising results with a combination of bevacizumab, a monoclonal antibody that inhibits VEGF, and the EGFR inhibitor erlotinib, that blocks these two important pathways in cancer growth [17].

Molecular characterization of CRC can guide therapeutic decisions or define patient prognosis. In fact, a classification based on four consensus molecular subtypes (CMSs) was established: (1) CMS1, associated with hypermutations, microsatellite instability and strong immune activation; (2) CMS2, the canonical subtype, characterized by an epithelial phenotype and increased WNT and MYC activation; (3) CMS3, characterized by metabolic dysregulation; (4) CMS4, which shows prominent transforming growth factor–β (TGFβ) activation, stromal invasion, and increasing angiogenesis [18]. Nonetheless, TNM staging remains the standard for clinical decision-making. In stage I and II, the disease is located in the colon or rectum, in stage III there is ganglionic spread and in stage IV there is already the presence of distant metastasis usually liver and lung [19]. Surgery is the optimal primary treatment for CRC and should be preceded by neoadjuvant radiotherapy. Chemotherapy can also be used in combination with radiotherapy for neoadjuvant treatment. Recurrence and metastatic patterns differ between colon and rectal cancers. Nevertheless, patient’s survival with metastatic CRC varies from 5 to 19 months, depending on disease stage. There is a clear need for new biomarkers predicting CRC behavior and innovative therapeutic targets [20,21].

Tribbles pseudokinase family members (*TRIB1* (C8FW or SKIP1), *TRIB2* (C5FW) and *TRIB3* (NIPK, SKIP3 or LKW)) might be biomarkers of interest in colorectal cancer. *TRIB1* lacks, while *TRIB2* and *TRIB3* have low, affinity for ATP, along with residual phosphotransferase capacity [22]. Instead of phosphorylating target proteins, they function as protein scaffolds that modulate diverse signaling cascades involved in proliferation, survival, differentiation and drug resistance [23]. Tribbles can lead to protein degradation by binding to ubiquitin ligases promoting the ubiquitylation of substrates [24], stabilization by suppressing sumoylation [25], or affecting signal transduction pathways through binding of signaling molecules like MEK and AKT [26]. For example, *TRIB2* has been previously identified as an oncoprotein that contributes to the pathogenesis of acute myelogenous leukemia (AML) through the inhibition of C/EBPα function, similarly to slbo inactivation by *Drosophila* Tribbles [27]. Different reports show that Tribbles proteins exert a role in the development of different types of tumors. However, they might exhibit an oncogenic or tumor suppressive behavior depending on the family member and cellular context [28]. For instance, *TRIB2* promoted AKT activation and, consequently, the inactivation of the transcription factor FOXO in melanoma. Expression of FOXO target genes, which can induce apoptosis, are therefore attenuated by *TRIB2* overexpression [29,30]. By contrast, *TRIB3* was shown to have the opposite effect [31], and its loss was associated with a more aggressive tumor phenotype [32]. Increased levels of *TRIB3* inhibited AKT activity and consequently induced cell death via FOXO in glioma cells [33]. These data support a scenario in which the balance between the expression of Tribbles might determine tumor progression, in this particular case through FOXO proteins activity via AKT. Several studies have been published reporting the role of Tribbles proteins in different types of cancer, including *TRIB1* in prostate [34], glioma [35], ovarian [36], and thyroid [37] cancers; *TRIB2* in melanoma [38], lung [39], liver [40,41,42], acute leukemias [27,43,44], and glioblastoma [45]; and *TRIB3* in lung [46], breast [47,48], renal [49], gastric [50], liver [51], retinoblastoma [52], glioblastoma [53], and ovarian [54] cancers; as well as all Tribbles members in colorectal cancer, as herein fully presented. Tribbles pseudokinases may have an immediate impact in cancer treatment being potential therapeutic targets.

**Figure 1:**
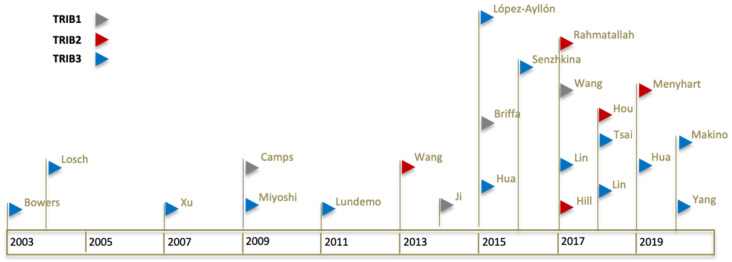
Timeline of Tribbles publications in colorectal cancer. Chronological representation of each significant publication. Each publication is represented by a coloured flag symbol (▷). Flag colours are associated with a different Tribble family member (*TRIB1* in grey flag, *TRIB2* in red flag, and *TRIB3* in blue flag). First author last name is shown for each flag. A total of four articles studying or including *TRIB1*, five for *TRIB2*, and 14 for *TRIB3*, in colon cancer, were identified from 2003 to 2020 and are included in the review.

With this review, we established a comprehensive timeline describing what is known about the putative role of each Tribble family member in the development of colorectal cancer (Figure 1). This document should also serve as a guide for future research, describing the available bioinformatic datasets regarding colorectal cancer studies.

## 2. Tribbles Amplification and Overexpression in Colon Cancer Tissues

The identification of increased genomic copy number (genomic amplification) in chromosome 8, combined with altered gene expression (up-regulation), allowed the first identification of *TRIB1* as a putative biologically relevant oncogene, in primary colorectal tumor or established colorectal cancer (CRC) cells lines containing the amplicon [55]. Half a decade later, a study published in 2014, identified *TRIB1* as a double minute (DM)-carried gene in a human CRC cell line [56,57]. DMs are small-paired, autonomously replicating, extrachromosomal DNA segments that contain amplified oncogenes, mainly present in solid tumors and identified in many human cancer cell lines [58]. Indeed, along with MYC and FGFR2 (among others), *TRIB1* was amplified in NCI-H716 cells compared to normal human peripheral blood DNA. Though the authors also showed elevated *TRIB1* mRNA levels in CRC cells, it was compared to non-paired normal colon tissue [56]. In a different study, *TRIB1* region was amplified in 7 out of the 15 CRC cell lines tested. *TRIB1* was clearly amplified and very highly expressed in NCI-H716 cells. However, there was no correlation between DNA copy number of the *TRIB1* region (Chr8: 126,393,571–126,567,050) and *TRIB1* protein. The authors did find a weak correlation between *TRIB1* copy number and mRNA expression [59]. They also found that *TRIB1* copy number was gained in 11% of primary CRC samples (*n* = 881 patients’ cohort, Oncomine database). From a different cohort, 11 tumors (14.4% out of 76 cases) were *TRIB1*-amplified; this value was above MYC amplification, for which only 6 tumors were amplified (7.4%) [59]. It was not until 2017 that the *TRIB1* gene was shown to be amplified and overexpressed in CRC tissues, when compared with paired surrounding non-tumor tissues from the same patients [60]. By using the TCGA database, the authors described that the copy number of *TRIB1* in human CRC tissues (*n* = 212) was significantly increased, when compared with normal colon tissues (*n* = 79). Data available from the Gene Expression Omnibus (GEO) [61], revealed that *TRIB1* gene expression levels in CRC tissues were elevated, compared with normal colon tissues (microarray expression studies from Oncomine) [60]. The authors experimentally validated the data by western blotting and found that the levels of *TRIB1* protein were elevated in 6 out of 8 (75%) CRC tumors when compared to matched adjacent non-tumor tissue. In addition, *TRIB1* protein overexpression was detected in 52 out of 75 (69.3%) of CRC cases as assessed by immunohistochemistry (IHC) compared with matched normal tissue [60].

The first hint that *TRIB2* expression was related to colon cancer was reported in 2013, when *TRIB2* protein was detected in CRC samples using tissue microarray analysis (TMA) by IHC [41]. Later in 2017, *TRIB2* mRNA and protein expression were found to be significantly increased in primary colon cancer tissue samples when compared to matched normal tissue samples [30]. However, none of these studies specifically focused on *TRIB2* in colon cancer. One year later, it was further confirmed that *TRIB2* was overexpressed in colorectal cancer patients, in primary tumor samples obtained from surgical resection, prior to receiving any kind of chemotherapy or radiotherapy [62]. The authors included an initial GEO database analysis, and found that *TRIB2* was highly expressed in primary tumor tissues compared with adjacent normal tissues [62]. These data have been experimentally validated by RT-PCR, showing that *TRIB2* gene expression was higher in a great proportion of tumors (73.3%), when compared to adjacent tissues. Accordingly, IHC staining showed that the average expression level of *TRIB2* was elevated in CRC tissues compared with normal tissues. Further paired analysis also demonstrated that *TRIB2* protein level was higher in tumors (76.7%) [62]. All these findings suggest that *TRIB2* is highly expressed in a large subset of CRC patients.

Though initially stated as “data not shown”, the very first input on *TRIB3* gene overexpression in colon tumor samples, when compared to matched normal colon samples, was published in 2003 [63], much earlier than the other two Tribbles family members (Figure 1). The authors had identified SKIP3, a protein with very high homology to other previously reported mammalian orthologs of Drosophila tribbles [63], later named *TRIB3*. While in the normal tissues tested (including colon), *TRIB3* mRNA levels were preferably elevated in liver, the highest expression was observed in tumor-derived cell lines, which included the colorectal adenocarcinoma SW480 cells [63]. *TRIB3* transcript levels were increased in colon adenocarcinoma samples, when compared to normal human tissues. Its tumor-specific localization was further confirmed by in situ hybridization [63]. In human tumor xenografts with HT-29 colorectal adenocarcinoma cells, *TRIB3* expression pattern was proximal to a region of cell death, localized to a periapoptotic region. Nonetheless, its localization did not correlate with cell proliferation nor with cells undertaking apoptosis [63]. The following year, another article showed elevated gene expression of *TRIB3* in different tumor cell lines [64]. In agreement with the previous report [63], one of the strongest signals was observed in the colorectal adenocarcinoma cell line SW480 [64]. Although not shown, the authors described a significant higher *TRIB3* gene expression in colon-carcinoma compared to the corresponding normal tissues. Moreover, they also observed up to 400-fold overexpression of *TRIB3* mRNA in human tumor tissues from primary tumors with different TNM stages versus normal colonic mucosa [64]. In 2007, a study on the *TRIB3* putative role in tumorigenesis, included the analysis of *TRIB3* mRNA expression level in different carcinomas [65]. *TRIB3* was highly expressed in colon tumor tissues, moderately in lung and esophageal, very limited in stomach, but not increased in liver or kidney cancers [65]. Interestingly, the authors discovered that *TRIB3* was overexpressed in all the colon cancer tissue pairs analyzed [65]. Consolidating the results described in previous studies for SW480 cells [63,64], it was shown that all other gastrointestinal cell lines tested (Caco2, DLD-1, LoVo, HCT116, HT-29, and KM12SM) expressed *TRIB3* gene [66]. Moreover, almost 90% of primary CRC samples analyzed had higher levels of *TRIB3* mRNA in tumors, compared to the paired normal regions [66]. *TRIB3* protein localized both in the nucleus and the cytoplasm of epithelial cells, while very weakly detectable in stromal cells [66]. The expression of *TRIB3* protein was positively correlated with its mRNA expression, though not all of the analyzed cases showed higher *TRIB3* levels in tumor regions than in normal regions [66]. Consistently with previous results herein described for colon, but not concerning liver [65], an additional report showed high levels of *TRIB3* in both colon cancer, hepatocellular carcinoma (HCC), and lung cancer tumor tissues, when compared with adjacent non-tumor tissues [67]. In agreement with the early studies, where *TRIB3* transcript levels were evaluated, high *TRIB3* protein levels were also found in SW480 cells, and those were associated with *TRIB3* mRNA expression [68]. Intriguingly, it was found that two *TRIB3* spliced isoforms were overexpressed in colon tumors [69]. The alternatively spliced transcripts uc002wdm/NM_021158 and uc002wdn/AK297546 showed an approximately 10- and 4-fold average increase, respectively, in colorectal cancer specimens, compared to matched morphologically normal tissues [69].

According to these data, it is unquestionable that all members of the Tribbles family are overrepresented in colon cancer and representative colorectal cancer cell lines (Table 1), which might represent useful biomarkers for disease detection. However, it remains to be investigated whether there is a correlation between *TRIB1*, *TRIB2* and *TRIB3* expression in these samples. To the best of our knowledge, there are no studies where the authors simultaneously analyzed two or more Tribbles in the same samples.

## 3. Clinical Significance of Tribbles Overexpression in Colon Cancer

To better understand the clinical impact of Tribbles pseudokinases overexpression in patients with colon cancer, several clinical parameters and clinicopathological features were analyzed and correlated to each member of the Tribbles family level in different samples. With regards to *TRIB1* and survival, one initial study did not find statistical differences in survival between patients with or without *TRIB1* (nor with MYC) gene amplifications [59]. However, a different study found that high (versus low) *TRIB1* gene expression correlated with poor survival in CRC patients [60]. Moreover, *TRIB1* protein overexpression was positively associated with distant metastasis and advanced staging [60].

For both melanoma and colon cancer patients, increased levels of *TRIB2* gene expression correlated with a significantly worse clinical outcome, which was not observed for pancreatic tumor patients [30]. In agreement, *TRIB2* levels in colorectal cancer were inversely correlated with survival rate of CRC patients, and positively correlated with tumor grade [62]. Patients with high expression of *TRIB2* experienced not only a worse overall survival, as more recurrence [62].

A recent report analyzed the top ten most frequently mutated genes, which mutations result in a disruptive protein structure [70]. From these, the authors identified the most significantly upregulated genes which correlated with the worst survival outcome. Finally, from these, they selected those genes which were theoretically druggable targets [70]. Using this elegant strategy, it was shown that the mRNA levels of *TRIB2* (as well as of DUSP4, dual-specificity MAPK phosphatase (4) were significantly higher in ACVR2A mutant colon cancers compared to wild-type cases [70]. ACVR2A, which encodes activin receptor type IIA, is a component of the TGFβ signaling pathway, shown to function as a tumor suppressor gene in human CRC-derived organoids [71]. Though ACVR2A mutations, reported in several cases of CRC [72], have been previously linked to earlier tumor stages (stages I/II) [73], *TRIB2* correlation to tumor staging was not performed within this study [70]. In an independent clinical cohort, high *TRIB2* expression was associated with worse recurrence-free survival (RFS), and also linked to mutations in ACVR2A gene [70].

In colonic premalignant polyps, *TRIB2* expression was associated with an increased risk for progression to colon cancer [74]. Along with phosphoinositide-3-kinase regulatory subunit 3 (PIK3R3) and poly(ADP-ribose) polymerase-14 (PARP14) (among others), *TRIB2* was up-regulated in sessile serrated polyps (SSA/Ps), while expressed at a lower level in both the right colon and hyperplastic polyps (HPs) samples [74]. Improved disease-free survival (DFS), though not OS, were, however, previously described in colorectal patients who expressed higher levels of PIK3R3 [75]. PARP14, by contrast, has been proposed as a novel drug target for different types of cancer, though colon cancer not yet included [76]. Despite sharing histological similarities with the typical hyperplastic polyps (HPs) [77], the SSA/Ps lesions are more prone to develop into cancer [78], which makes this finding clinically relevant and proposes *TRIB2* as a useful biomarker to early distinguish colonic lesions [74].

*TRIB3* expression was highest at Duke’s stages B > A (which represents stages with no or infrequent dissemination) > C > D (representing more invasive and metastatic stages), suggesting an inverse correlation between invasion and progression and *TRIB3* expression. Nevertheless, although the lowest level was observed in stage D, it was still much higher than in normal colorectal epithelium [64]. Such as for *TRIB1* [60], metastasis (M0/M1) was correlated with *TRIB3* expression [66]. However, metastatic sites, tumor size, invasion, lymph node metastasis or lymphatic and venous invasion, were not significantly correlated with *TRIB3* levels [66]. Higher expression of *TRIB3* was inversely correlated with overall survival rate in two independent studies [66,67]. Unquestionable, high *TRIB3* expression levels correlated with low survival rates and poor outcomes of patients with CRC [79].

Recently, two independent predictive models included *TRIB3* expression levels [80,81]. The analysis of the association between differentially expressed genes and overall survival in a set comprised with 347 colon (but not rectal) cancer patients with a follow-up time greater then 90 days, from TCGA dataset, identified *TRIB3* as a high-risk RNA, along with BDNF, KLF4, SESN2 and SMOC1 [80]. Based on the significant correlation of the 5 genes expression with tumor status and tumor stage, the authors developed a predictive multi-mRNA-based model for overall survival, assigning risk scores to each patient and obtaining a prognosis tool, aiming treatment optimization for patients [80]. These results reinforced the usefulness of *TRIB3*, though combined with other genes, as a potential prognostic biomarker for colon cancer. On the other hand, *TRIB3* was associated, together with other immune-related genes such as CHGA, LGALS4, LEP, NOX4, IL17A, HSPD1, and CASP7, with colon cancer prognosis [81]. Based on the gene expression profiles, the authors developed a molecular classifier tool. It was validated in 277 samples from TCGA dataset that had available both gene expression and survival status and time, and in an independent validation dataset (213 colon cancer samples from GEO). Alongside the analysis of the tumor-infiltrating immune cells in colon cancer, a novel predictive tool for colon cancer prognosis, which included *TRIB3* levels, was established [81].

These results suggest a significant link between Tribbles expression and patient prognosis (Table 2). Specifically, *TRIB1* and *TRIB3* might be considered candidates as predictive markers of tumor metastasis development. Additionally, there is abundant data confirming the role of *TRIB2* in drug resistance functioning as a predictive biomarker for drug response. This is of utmost importance for the clinicians at the time of deciding the course of treatments.

## 4. Tribbles Gene and Protein Expression Regulation in Colon Cancer

**Figure 2:**
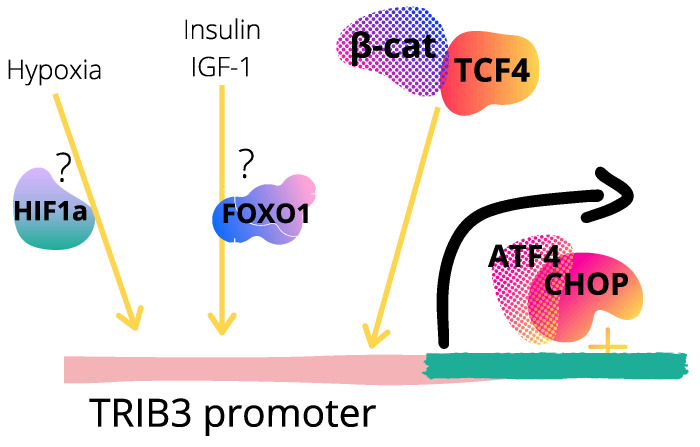
*TRIB3* upstream regulation in colorectal cancer. Proposed mechanisms regarding *TRIB3* regulation at the promoter level based on references [63,67,79,85,86] are shown. *TRIB3* is a transcriptional target of the ATF4–CHOP [87] and β-catenin–TCF4 [79] complexes through direct binding at specific sites at *TRIB3* genomic sequence, localized at +201 to +312 and at −12 to −3, respectively. *TRIB3* is proposed to be positively or negatively regulated by HIF1α or FOXO1 at the transcriptional level, respectively, in response to specific stimuli that modulate transcription factors merged (TF) activity such as hypoxia or feeding signals. TF are depicted as random elements from Canva design.

Recognizing how Tribbles are regulated in colon cancer is indispensable to better understand the physiopathology of this type of cancer and Tribbles involvement in the disease development and progression. While for *TRIB1*, gene amplification might partially support its increased levels not only in colon, but also in other types of cancer [82,83], the mechanism behind *TRIB2* overexpression in colon cancer patients is still unclear. In other tumor types, however, different mechanisms for *TRIB2* regulation have been described [41,84]. For instance, while Wnt signaling regulated *TRIB2* in hepatic cancer HepG2 cells, it had no effect in the LS174T CRC cell line [41]. This might reflect that different transcriptional programs are being differentially activated in different cancer types. By contrast, *TRIB3* expression was induced in CRC cells after β-catenin activation by Wnt3a, in a dose- and time-dependent manner. Conversely, decreased *TRIB3* expression was observed in response to β-catenin depletion [79]. The authors recognized *TRIB3* as a transcriptional target of the β-catenin–TCF4 complex and identified a TCF4-binding site (CACAGCTGCG motif) at the C-terminal domain of the *TRIB3* promoter region [79] (Figure 2). Moreover, β-catenin inhibited *TRIB3* degradation, contributing to the overall increased levels of *TRIB3* in CRCs by inducing its protein stability [79]. Additional mechanisms might be involved in *TRIB3* regulation in CRC. In HT-29 colorectal adenocarcinoma cells subjected to hypoxia, *TRIB3* was upregulated at the transcript and protein level [63], suggesting *TRIB3* could be regulated in CRC by classical hypoxia-related transcription factors, such as HIF1α (Figure 2). More recently, it was shown that *TRIB3* was up-regulated concomitantly to the induction of C/EBP-homologous protein (CHOP) in HCT116 cells, while down-regulated following CHOP knockdown [85,86], revealing *TRIB3* being downstream of CHOP-activated pathways (Figure 2), as previously proposed [87,88]. In addition, insulin and IGF-1 induced *TRIB3* protein expression in human colon cancer HCT-8 cells (Figure 2), as well as in hepatoma HepG2 and lung cancer A549 cell lines [67]. Interestingly, other C/EBP proteins have been linked to the oncogenic role of Tribbles proteins in myeloid cancers [89].

## 5. Tribbles Pharmacological Modulation in Colon Cancer

Genes that are over-expressed in cancer are more likely to be putative pharmacological targets [90]. Tribbles proteins have emerged as interesting novel therapeutic targets, with their unique pseudokinase domain providing a potential opportunity for drug design approaches [91,92]. To the best of our knowledge, there is lack of studies in colon cancer patients that identified Tribbles differential expression in response to approved cancer treatments.

Recent work demonstrated that afatinib, an approved irreversible electrophilic covalent EGFR/HER2 inhibitor for lung cancer treatment, increased *TRIB2* degradation in human acute myeloid leukemia (AML) cancer cells [93], demonstrating for the first time that *TRIB2* might be a druggable protein. Early this year, others showed similar results in human hepatoma cell lines, where afatinib treatment (10 µM, 20 h) reduced *TRIB2* protein levels and decreased cellular viability over 50% [94]. In SW-48, Colo205 and HT-29 CRC cell lines, though Tribbles levels were not investigated, afatinib treatment (1 or 10 µM, 48 h) also reduced cell viability [95], representing an opportunity for further studies, in order to establish *TRIB2* targeting as a potential strategy for treating colon cancer.

Regarding *TRIB3*, for which more studies have been performed, potential *TRIB3* druggability has been suggested, although not yet fully validated in the CRC setting. A study that analyzed different cell lines from the NCI60 database (using CellMiner), representing cancer types that typically receive treatment with erlotinib, such as breast cancer, colon cancer and NSCLC, identified *TRIB3* at the top three genes that showed the strongest correlation with EGFR inhibition [96]. The average expression of the seven genes identified (LCN2, MET, MMP7, PTPRZ1, *TRIB3*, UGT1A6 and COL17A1 was lower in more sensitive cells [96]. These results suggest that *TRIB3* might be a useful clinical predictive biomarker for erlotinib action in the absence of EGFR-activating mutations for colon cancer patients. Conversely, high expression of *TRIB3* could be related to CRC resistance to anti-EGFR therapies, and future research should clarify this potential relationship. Interestingly, it was recently shown that increased levels of *TRIB3* were associated with elevated EGFR stability and signaling activity in lung cancer, suggesting that the disruption of the *TRIB3*-EGFR interaction could be a novel therapeutic target [97]. Nevertheless, whether *TRIB3* might be in fact a downstream erlotinib therapeutic target still remains to be elucidated.

**Figure 3:**
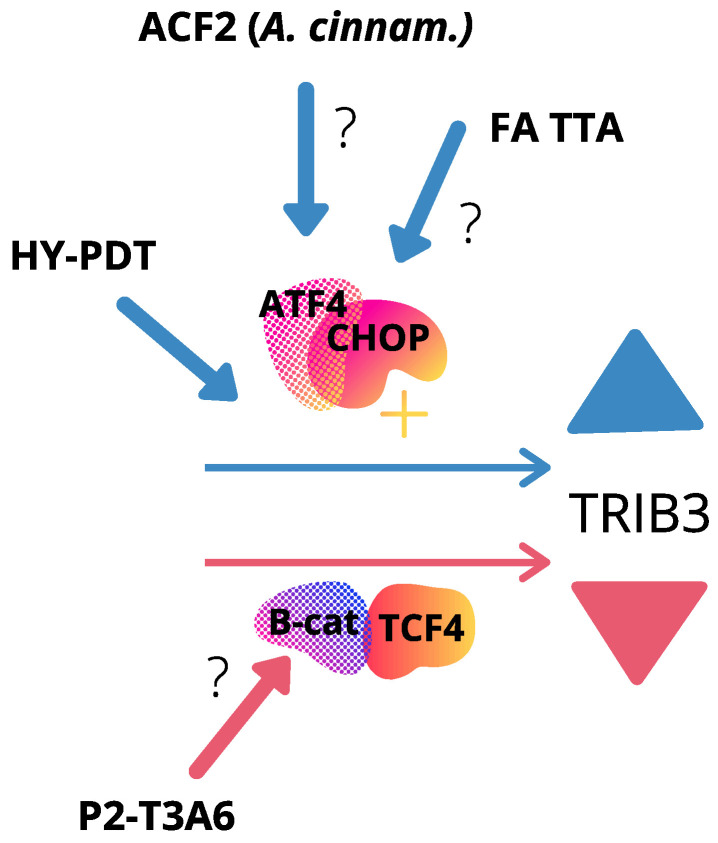
Proposed mechanisms for *TRIB3* pharmacological modulation in colorectal cancer. Compounds that alter *TRIB3* expression based in references [79,85,86,98], are depicted on figure. *TRIB3* was upregulated in response to FA-TTA and ACF2 extract treatments, along with CHOP induction. *TRIB3* levels were elevated after HY-PDT, in a CHOP-dependent fashion. P2-T3A6 treatment caused disruption of the β-catenin–*TRIB3* interaction, leading to decreased *TRIB3* levels. Symbols: ▲ represents up-regulation (in blue color) and ▼ represents down-regulation (in pink color) of *TRIB3*. Transcription factors are depicted as random elements from Canva design. FA-TTA, bioactive chemical modified fatty acid analog saturated 3-thiatetradecylthioacetic acid; HY-PDT, photosensitizer hypericin and photodynamic therapy; ACF2 is a specific extract from *Antrodia cinnamomea*, a native Taiwanese rare mushroom; P2-T3A6, fusion peptide.

Another study showed that transcript and protein levels of *TRIB3* were upregulated in SW620 cells treated with the bioactive chemical modified fatty acid (FA) analog saturated 3-thia FA tetradecylthioacetic acid (TTA), compared to non-treated cells [98]. TTA not only inhibited the growth of colon cancer cells, but also induced the expression of several other genes involved in endoplasmic reticulum (ER) stress and unfolded protein response (UPR), such as CHOP and C/EBPβ, whereas Cyclin D1 was down-regulated [98]. After treating HCT116 cells with the photosensitizer hypericin and photodynamic therapy (HY-PDT) [99], at the higher concentration range, *TRIB3* protein levels were induced, along with induction of CHOP, activation of the autophagy signal and cell death [85]. Nevertheless, *TRIB3* up-regulation was CHOP-dependent, reflecting a most likely indirect action of HY-PDT in *TRIB3* modulation [85]. Importantly, *TRIB3* has been previously located downstream of CHOP-mediated pathways [87]. Similarly, after treatment of HCT116 cells with a specific extract from *Antrodia cinnamomea*, a native Taiwanese rare mushroom, *TRIB3* transcript levels were induced in a dose-dependent manner [86]. Though CHOP itself was also up-regulated, none of the other downstream genes of the apoptosis and autophagy CHOP-mediated pathways were affected. Interestingly, this extract (ACF2) inhibited cellular viability in different human colorectal cancer cell lines such as HCT116, HT29, SW480, Caco-2 and Colo205 [86]. Though the authors validated that ACF2 inhibited the growth of CRC cells subcutaneously inoculated into athymic nude mice, the levels of *TRIB3* were not evaluated in the tumors [86]. In both primary CRCs and HCT-8 ileocecal adenocarcinoma cells, a fusion peptide named P2-T3A6, which inhibited cell viability and migration, demonstrated having binding affinity with *TRIB3*. As a result, *TRIB3* protein degradation was accelerated, together with inhibition of *TRIB3* gene transcription [79]. Interestingly, P2-T3A6 peptide caused disruption of the β-catenin–*TRIB3* interaction, though it did not affect other *TRIB3* known interactions, such as with SMAD3 [100], p62 [67] or AKT [31], which might reflect an indirect peptide action on *TRIB3* regulation by β-catenin [79], as previously described above. Though there is few evidence regarding pharmacological modulation of *TRIB3* in CRC, all cases most likely represent an indirect effect through β-catenin or CHOP pathways (Figure 3).

## 6. Genetic Modulation of Tribbles in Colon Cancer

It is challenging to distinguish between driver and passenger mutations. Reports on genetic alterations of Tribbles genes in human cancer are rare. A recent study described a novel fusion transcript derived from a chromosomal translocation between the *TRIB2* and the PRKCE genes, in pulmonary carcinoid tumors [101]. Nevertheless, to the best of our knowledge, no Tribbles mutations have been associated with CRC so far.

The first report on the effect of *TRIB1* downregulation in colorectal cancer cells was published in 2014, and showed that interfering with *TRIB1* (cells stably transfected with shRNA vectors, specifically knocking down *TRIB1* gene expression) led to the decrease of the number of double-minute chromosomes (DMs) formed, along with genomic instability and cytotoxic DNA damage in the NCI-H716 tumor cells [56]. Although not specifically tested for *TRIB1*, the authors showed that downregulation of other DM-carried oncogenes amplified along the same chromosome, such as MYC and FGFR2, led to impairments of cellular proliferation and invasion, suggesting a parallelism with *TRIB1* [56]. *TRIB1* stable or transient overexpression in SW480 and LoVo cells, respectively, showed enhanced migratory and invasion ability of the cells, compared with controls [60]. Moreover, SW480-*TRIB1* cells exhibited increased metastasis capacity, evaluated in vitro by cell adhesion to extracellular matrix (ECM) assays [60]. Conversely, silencing *TRIB1* expression by shRNA against *TRIB1*, in both SW480-*TRIB1* and COLO320HSR cells, reduced the level of cell migration and invasion [60], establishing a role for *TRIB1* in colon cancer cells motility.

Similar to the findings previously described for *TRIB1*, siRNA down-regulation of *TRIB2* in SW48 and LoVo CRC cells suppressed proliferation, induced cell cycle arrest (increased G0/G1-phase ratios along with reduced the S-phase ratios) and promoted cellular senescence [62]. Although cell growth was affected, *TRIB2* knock-down did not induce apoptosis. The role of *TRIB2* in the cancer progression phenotype was further confirmed in these cells, since both proliferation and cell cycle progression were accelerated by *TRIB2* overexpression, along with decreased rates of cellular senescence [62].

In 5 different CRC cells (DLD-1, LoVo, HCT116, KM12SM and SW480), *TRIB3* knock-down was obtained by siRNA [66]. For all cell lines, silencing of *TRIB3* correlated with decreased cellular proliferation. A very robust effect was observed at day 4 after plating, mainly in DLD-1, LoVo, and HCT116 *TRIB3*-depleted cells [66]. The first in vivo experiment regarding Tribbles cellular genetic manipulation was reported in 2015, where the authors aimed to evaluate the role of *TRIB3* in CRC tumorigenesis [67]. Genetically diabetic KK-Ay mouse, a model of human T2D, showed not only higher *TRIB3* expression in the liver and lungs, but also the xenografted tumors from the diabetic model displayed elevated *TRIB3* expression, when compared with C57BL/6 mice [67]. After being inoculated in BALB/c nude mice, *TRIB3* knock-down in HCT-8 CRC cells led to decreased metastasis and growth, revealing an antitumor role of *TRIB3* silencing. A coincident *TRIB3*-dependent phenotype was observed for human hepatoma HepG2 cells [67]. These results might be particularly important for T2D patients, who are at greater risk to develop liver or colorectal cancers [102]. The first in vivo modulation of *TRIB3* was reported in 2019. Few years later from previous report, the same research group down-regulated *TRIB3* expression in C57BL/6J-ApcMin/J mice fed a high-fat diet (Trib3-KD), aiming to specifically explore the role of *TRIB3* in intestinal tumorigenesis [79]. Despite no differences in weight changes between groups, Trib3-KD mice revealed higher survival rate and no colon or small intestinal tumors at the 10 weeks of age time point. By contrast, 75% and 50% of control mice developed tumors within their colons and small intestines, respectively [79]. Interestingly, mice overexpressing *TRIB3* (*TRIB3*-OE) showed a slower increase in the high-fat diet-induced body weight gain, despite the shorter colon length. As anticipated, *TRIB3*-OE mice showed a heavier tumor burden, reflected by a decrease in respective survival rates [79].

In colorectal cancer, the epithelial to mesenchymal transition (EMT) is associated with a more invasive or metastatic phenotype, as cells show increased motility (mesenchymal properties), while losing their epithelial characteristics [103]. A very recent report showed that down-regulating *TRIB3* in different colon carcinoma cell lines, such as SW480, HCT116, CaCO_2_, SW48 and SKCO1, led to a mesenchymal-epithelial transition (MET) phenotype [104].

It is evident that there is still much more to be learned about the role of these proteins in the process of cancer progression. What we do know so far, is that the majority of the reports pinpoint Tribbles as oncogenes in several cancer types, including colon cancer (Table 3). This suggests that modulation of Tribbles protein levels might have a positive impact delaying the oncogenic process. It is still to be determine if down-regulation of each Tribbles protein individually would trigger a positive feedback on any of the other Tribbles.

## 7. Downstream Mechanisms Regulated by Tribbles in Colon Cancer

A better understanding of the downstream pathways, potentially regulated by Tribbles proteins, is essential to better direct the therapeutic options available, based on Tribbles expression and the mechanism of action involved. Though an association does not always reflects causation, analyses from tissue microarray consisting of 118 Dukes’ A and B CRC patients revealed a significant correlation of *TRIB1* protein expression with ERK signaling pathway activation, and AKT and MYC protein abundance [59]. In agreement, *TRIB1* overexpression (*TRIB1*-OE) in SW480 cells also increased ERK, as well as Src and FAK (focal adhesion kinase), protein phosphorylation [60]. In addition, *TRIB1* overexpression led to up-regulation of MMP-2 and MMP-9 proteolytic enzymes protein levels. Conversely, RNA interference *TRIB1* silencing in *TRIB1*-OE cells fully reversed the previous phenotype [60]. Moreover, specific ERK, Scr and FAK inhibitors independently down-regulated MMP-2 (but not MMP-9) expression in *TRIB1*-OE cells [60].

In *TRIB2* knocked-down SW48 and LoVo cells, increased p21 protein and mRNA was observed, while p53 (in contrast with previous report in a different cancer type, ie, melanoma [30]), cyclin D1 or p16 levels remained unchanged [62]. Conversely, overexpression of *TRIB2* showed the inverse phenotype (i.e., decreased p21), in a p53-independent manner. The authors demonstrated *TRIB2* negatively regulated p21 at the promoter level, through *TRIB2* kinase-like domain binding and cooperation with AP4, which was elevated in CRC tumor compared with the corresponding normal tissues [62]. Though *TRIB2* did not directly influence AP4 expression, upon *TRIB2* over-expression, AP4 protein was further enriched at the p21 promoter [62]. The observed cellular events were validated in a CRC xenograft nude mice in vivo model, subcutaneously injected with stable *TRIB2*-knockdown SW48 cells, where p21 expression levels were significantly increased, compared to controls [62].

*TRIB3* has been previously described, in other cellular types, to negatively regulate Akt-mTOR pathway by direct binding to AKT and consequent dephosphorylation [31]. In agreement, depletion of *TRIB3* in HCT116 cells reversed the reduction of phospho-Akt and phospho-mTOR in response to *A. cinnamomea*-derived extract ACF2 treatment [86]. Intricately, *TRIB3* silencing also decreased ACF2-induced LC3-II levels [86]. Making use of bioinformatic analysis, Hua et al. identified an enrichment of the Wnt signaling gene set in *TRIB3*-overexpressing cells in patients with CRC [79]. Experimentally, the authors verified that β-catenin transcriptional activation correlated with *TRIB3* levels in different CRC cell lines. Moreover, when comparing tumor with adjacent normal tissues, higher *TRIB3* and β-catenin expression, along with proteins co-localization, was observed. By contrast, colon tissues from *TRIB3*-KD mice showed much lower expression of genes regulated by Wnt signaling to β-catenin, compared to control mice, such as *Axin2*, *c-Myc*, *Cyclin D1*, and *TRIB3* itself. *TRIB3* and TCF4 were shown to interact, while depletion of *TRIB3* decreased the formation of the β-catenin-TCF4 complex. Finally, it was demonstrated that the up-regulation of these genes regulated by Wnt, in response to *TRIB3* overexpression, was β-catenin dependent, as cellular β-catenin depletion reversed the phenotype [79]. In the context of colon cancer, all three Tribbles seem to induce signaling pathways that are commonly associated with tumor progression.

## 8. Conclusions and Future Perspectives

There has been accumulating evidence that all members of the Tribbles family play a role in tumorigenesis. Whether they contribute to cancer progression or impairment is still an open question. This review highlights the existing research on the contribution of *TRIB1*, *TRIB2* and *TRIB3* to colorectal cancer as well its potential as biomarker for disease progression and/or prognosis. There is now accumulating data that *TRIB2* acts as an oncogene in several tumor types, including colon cancer. This is not as clear for *TRIB3*, as it was found that *TRIB3* overexpression negatively regulates the mTOR pathway, which is hyperactivated in several tumor types. On the contrary, different studies show that *TRIB3* overexpression is positively correlated with poor prognosis and survival of colon cancer patients, highlighting the potential of using *TRIB3* as a predictive biomarker. These data suggests that Tribbles family members role might be cell context dependent. Moving forward, it will be important to thoroughly study the interaction of all family members as they might have redundant roles in specific contexts. The generation of in vivo knock-out models using CRISPR technology would certainly contribute to decipher the role of each Tribble isoform in CRC development. On the other hand, colon cancer stem cells (CCSC) have been associated with patient relapse and chemoresistance, with diverse molecular mechanisms involved [105]. Importantly, high Tribbles levels have been linked with quiescent stem cell population, at least in hematopoietic diseases [106]. Thus, it is of great interest to understand the role of this family of proteins in the maintenance of CCSC and how they contribute to drug resistance. Furthermore, as new data emerge suggesting that this family of pseudokinases might be therapeutically modulated, it is of outmost importance to thoroughly understand the mechanisms by which these proteins are governed. This will potentiate the development of novel pharmacological approaches or the repurposing of existing drugs that specifically target the Tribbles proteins and improve patient care.

## Figures and Tables

**Table 1:** Tribbles amplification and overexpression in colon cancer tissues. Main result obtained for each study included in the section is shown for each Tribble member. Details of the samples, databases and methodology used by the authors is detailed under respective column. Whenever available, the number of samples analyzed is specified under *n*. nd, not defined.

Tribbles	Main Result	Samples		Databases/Experimental Methods	Author, Year (Reference)
		Disease	Control		
***TRIB1***	Genomic amplification; Increased copy number.	Primary colorectal tumors (*n* = 31); CRC cell lines: DLD-1, HCT116, p53HCT116, SW48, LoVo, SW480, SW837, HT-29, T84, Colo 201, Colo 320DM, LS411N, SK-CO-1, NCI-H508, NCI-H716 (from ATCC).	Normal colon RNA isolated postmortem from different donors without a history of colorectal cancer (*n* = 5) (from Ambion).	Agilent Oligonucleotide Array-Based CGH for Genomic DNA Analysis; Oligonucleotide-based Human Genome Microarrays (Agilent Technologies).	Camps, 2009 [55]
	Genomic amplification; DM-carried gene; 5 × mRNA increase.	NCI-H716 cell line.	Normal human peripheral blood; Non-paired normal colon tissue.	Immunoblotting analysis with anti-*TRIB1* antibody (Abnova); Affymetrix GeneChip Human Mapping SNP6.0 array (analysis of DNA copy number changes); qRT-PCR.	Ji, 2014 [56]
	Genomic amplification in 7 out of the 15 cell lines tested; 11–14.4% gain in CRC samples.	DLD-1, HCT116, HCT116p53-/-, SW48, LoVo, SW480, SW837, HT29, T84, Colo 201, Colo 320DM, LS411N, SK-CO-1, NCI H508 and NCI H716 cells (ECACC or ATCC); primary CRC samples (*n* = 881); CRC tumors (*n* = 76).	nd	Oncomine database; Comparative genomic hybridization (CGH) NimbleGen microarray (Roche): GSE72296; Illumina Whole Genome Gene Expression Profiling: GSE72544; Fluorescence in situ hybridisation (FISH): *TRIB1*/CEN8p probe (Abnova); Tissue microarray (TMA), automated quantitative analysis (AQUA) with anti-*TRIB1* rabbit polyclonal antibody.	Briffa, 2015 [59]
	Genomic amplification; Increase in mRNA and protein levels.	(1) Human CRC tissues (*n* = 212); (2) CRC (*n* = 70); (3) Colon adenocarcinoma (*n* = 4(1); (4) CRC tumor (*n* = 8).	Normal colon tissues: (1) *n* = 79; (2) *n* = 12; (3) *n* = 5; (4) paired surrounding non-tumor tissues (*n* = 8).	Oncomine: (1) TCGA; GEO: (2) GSE9348; (3) GSE5206 (GEO); (4) Western blot & IHC (protein levels).	Wang, 2017 [60]
***TRIB2***	Detection in CRC samples.	Human CRC samples.	nd	Human CRC tissue microarray slides (Biomax, Genvelop or UMass Cancer Center Tissue Bank).	Wang, 2013 [41]
	Increase in mRNA and protein levels.	Primary colon cancer tissue (*n* = 14).	Adjacent paired non-tumor colon samples (*n* = 14).	Immunoblot analysis.	Hill, 2017 [30]
	(1) Higher protein levels; (2) overexpressed in 73.3% of tumors.	(1) CRC patients samples; (2) Primary tumor (*n* = 186); (3) Adenocarcinoma (*n* = 45)	(1) Normal colorectal tissues (15 pairs); Normal colon: (2) *n* = 54; (3) *n* = 34.	(1) RT-PCR; IHC staining; GEO: (2) GSE41258; (3) GSE20916.	Hou, 2018 [62]
***TRIB3***	Gene and protein overexpression.	(1) Colon tumor; (2) SW480 cells; (3) Xenografts with HT-29 cells.	(1) Matched normal colon samples.	Northern blot analysis; Real-time PCR; In situ hybridization.	Bowers, 2003 [63]
	Gene overexpression.	(1) SW480 cells; (2) Tumor (*n* = 241); (3) human tumor tissues from primary cancers with different TNM stages (*n* = 24).	(1) nd; (2) Corresponding normal tissues from individual patients; (3) Normal colonic mucosa (*n* = 24)	Cancer profiling array; LightCycler PCR technology.	Lösch, 2004 [64]
	Overexpression.	Colon tumor tissues.	Colon pairs (*n* = 11)	qRT-PCR; data not shown.	Xu, 2007 [65]
	Gene and protein overexpression.	(1) Caco2, DLD-1, LoVo, HCT116, HT-29, KM12 SM, SW480 cells; (2) Primary CRC specimens (*n* = 202)	(1) nd; (2) Adjacent normal colorectal mucosa (*n* = 202)	IHC staining; Gel RT-PCR; Real-time RT-PCR.	Miyoshi, 2009 [66]
	Protein overexpression.	Colon tumors (*n* = 76)	Adjacent non-tumour tissues of the human colon (*n* = 72)	Tissue microarray (TMA); Immunohistologic staining.	Hua, 2015 [67]
	Gene and protein overexpression.	SW480 cells	-	Western blot; gel RT-PCR.	Lin, 2018 [68]
	Alternative transcripts overexpression	Colorectal cancer specimens (*n* = 40)	Normal tissues matched morphologically (*n* = 40)	TCGA RNA-Seq datasets; Gene Ontology database.	Snezhkina, 2016 [69]

**Table 2:** Clinical significance of Tribbles overexpression in colon cancer. Main results regarding the correlation between the expression of each Tribbles family member and its clinical impact. Sample details, databases and methodology used by the authors is detailed under the respective column.

Tribbles	Main Result	Samples/Databases/Experimental Methods	Author, Year (Reference)
***TRIB1***	No association with survival.	Dukes’ A and B stages CRC patients (*n* = 118); Tissue microarray.	Briffa, 2015 [59]
	Lower Disease Free or Specific Survival.	CRC patients; PrognoScan tool and gene expression databases (GSE17536: *n* = 145, GSE14333: *n* = 226 and GSE17537: *n* = 49); GEO database-GSE17537; anti-*TRIB1* ICH correlation with clinicopathological features.	Wang, 2017 [60]
***TRIB2***	Worse clinical outcome.	Gene Expression Omnibus (GEO) database: GSE17536	Hill, 2017 [30]
	Worse overall survival (OS); more recurrence; higher tumor grade.	Gene Expression Omnibus (GEO) databases: GSE21510; GSE25071; GSE17536. The Cancer Genome Atlas (TCGA).	Hou, 2018 [62]
	Worse survival outcome in ACVR2A mutant colon cancers; Worse recurrence-free survival.	Independent clinical cohort (formalin-fixed paraffin-embedded (FFPE) cancer tissues from 171 CRC patients; GEO datasets with published survival times (GSE17538, GSE12945, GSE31595, GSE14333, GSE37892, GSE33114, GSE41258, GSE39582, GSE30540, GSE18088, GSE26682, and GSE1329(4); Drug–Gene Interaction database DGIdb 3.0; TCGA repository.	Menyhart, 2019 [70]
	Up-regulation in cancer-prone sessile serrated polyps.	Human colonic premalignant polyps: sessile serrated polyps (SSA/Ps), right colon and hyperplastic polyps (HPs) samples (GEO datasets: GSE10714, GSE45270, GSE76987, GSE4384(1).	Rahmatallah, 2017 [74]
***TRIB3***	Lower expression in more invasive/metastatic stages (stage D).	Dukes’ A, B, C and D stages CRC patients.	Lösch, 2004 [64]
	Metastasis (M0/M(1) correlation; inverse correlation with OS.	Primary CRC specimens and adjacent normal colorectal mucosa from 202 patients who underwent surgery for CRC (Kyusyu University, 1992–2002; Osaka University, 2002–2006).	Miyoshi, 2009 [66]
	Inverse correlation with OS.	Surgically removed human primary colon carcinoma and normal colon tissue specimens (Alenabio, Xian, China).	Hua, 2015 [67]
	Low survival rate; poor outcomes.	CRC dataset from The Cancer Genome Atlas (TCGA). GEO dataset: GSE41258.	Hua, 2019 [79]
	Significant correlation with tumor status & stage.	Colon cancer patients with a follow-up time greater then 90 days (*n* = 347); TCGA dataset.	Huang, 2019 [80]
	Association with prognosis.	277 samples (GSE17538); Gene expression profiles (TCGA).	Yang, 2020 [81]

**Table 3:** Tribbles genetic modulation response in colon cancer. Main results obtained for cellular genetic manipulation of Tribbles is described, separately for either up- or down-regulation, and respective reference (author, year). Not applicable (na) is shown when the data was not available.

Tribbles	Genetic Manipulation		Author, Year (Reference)
	Up-Regulation	Down-Regulation	
***TRIB1***	na	Decrease in DM formation; genomic instability; cytotoxicity (NCI-H716 cells).	Ji, 2014 [56]
	Increased migration, invasion and metastatic capacity (SW480 and LoVo cells).	Reduced migration and invasion (SW480-*TRIB1* and COLO320HSR cells).	Wang, 2017 [60]
***TRIB2***	Decreased cellular senescence, increased proliferation and cell cycle progression.	Decreased proliferation, induced cell cycle arrest and cellular senescence (SW48 and LoVo cells)	Hou, 2018 [62]
***TRIB3***	na	Decreased proliferation (DLD-1, LoVo, HCT116, KM12SM and SW480 cells).	Miyoshi, 2009 [66]
	na	Decreased metastasis and growth (HCT-8 cells in nude mice).	Hua, 2015 [67]
	Decreased survival rate and tumor burden (*TRIB3*-OE mice).	Increased survival rate, no colon tumor development (Trib3-KD mice).	Hua, 2019 [79]
	na	Mesenchymal-epithelial transition phenotype (SW480, HCT116, CaCO_2_, SW48 and SKCO1 cells).	Makino, 2020 [104]
